# The Role of Astrocytes in the Inflammatory Response in Neurodiseases

**DOI:** 10.1002/mco2.71069

**Published:** 2026-10-19

**Authors:** Shijun Peng, Lepeng Wang, Jia Ouyang, Ruen Liu

**Affiliations:** ^1^ Department of Neurosurgery Peking University People's Hospital, Xicheng District Beijing China

**Keywords:** astrocytes, glial cell crosstalk, neuroinflammation, neurological diseases, therapeutic targeting

## Abstract

Astrocytes have emerged as central regulators of neuroinflammation, extending far beyond their traditional roles as structural and metabolic support cells. In neurological disorders, astrocytes undergo reactive remodeling that encompasses morphological, transcriptional, metabolic, and functional changes. Rather than fitting into a fixed A1/A2 dichotomy, reactive astrocytes exist along a disease‐, region‐, and stage‐dependent continuum shaped by local pathology and intercellular crosstalk. This review summarizes how astrocytes contribute to neuroinflammatory processes in Alzheimer's disease, Parkinson's disease, amyotrophic lateral sclerosis, Huntington's disease, central nervous system injury, infection, multiple sclerosis, epilepsy, and autism spectrum disorder. We highlight convergent mechanisms, including cytokine and chemokine release, complement activation, glutamate and ion homeostasis disruption, oxidative stress, metabolic reprogramming, and blood‐brain barrier regulation, while emphasizing disease‐specific astrocytic programs. We further discuss astrocyte interactions with microglia, neurons, oligodendrocytes, endothelial cells, and peripheral immune cells, as well as key regulatory pathways, epigenetic remodeling, and lipid‐dependent metabolic signaling. Finally, we evaluate current and emerging astrocyte‐targeted therapeutic strategies, including metabolic modulation, receptor targeting, gene editing, nanodelivery, and gut–brain axis interventions. Defining functionally validated astrocyte states and developing biomarker‐guided, stage‐adaptive therapies may enable more precise intervention in neuroinflammatory and neurodegenerative diseases.

## Graphical abstract

Schematic representation of key astrocytic pathways contributing to pathology in the pathogenesis of AD, PD, ALS, and HD. In AD, astrocytes exhibit Aβ plaque‐associated activation characterized by GFAP/S100β upregulation, accompanied by impaired EAAT2‐mediated glutamate clearance and apoE4‐induced BBB disruption. In PD, α‐synuclein pathology drives STAT3/NF‐κB activation, leading to JAK/STAT3‐mediated neuroinflammation and NR4A2 downregulation‐mediated loss of neuroprotection. ALS‐associated SOD1 mutations induce astrocytic EAAT2 dysfunction and AMPAR/GluR2 dysregulation, while HD pathogenesis features mHTT‐mediated EAAT2/Kir4.1 suppression and HMGB1‐TLR/RAGE‐driven neurotoxicity. The figure highlights the central, yet diverse, roles of astrocytes across major neurodegenerative diseases.

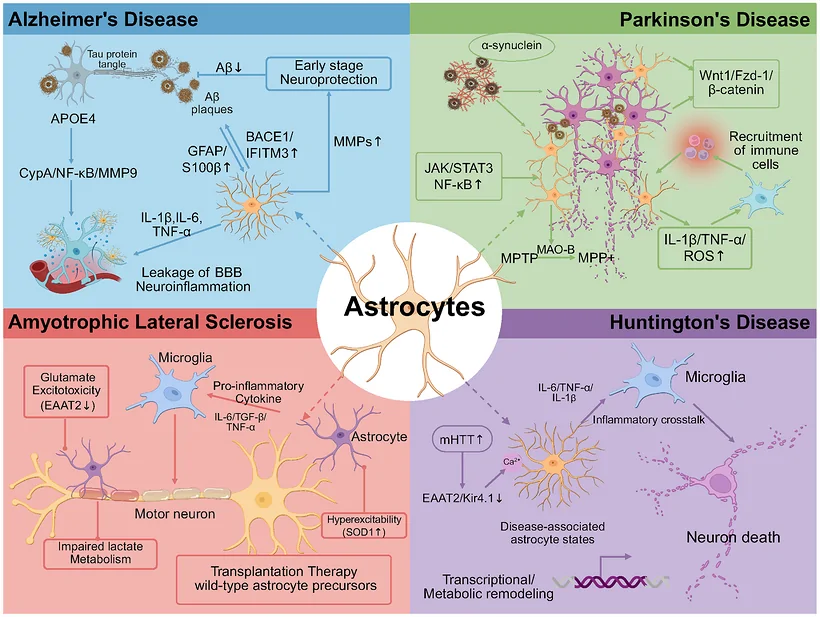

## 1 Introduction

The central nervous system (CNS) has long been regarded as an “immune‐privileged” site. However, recent research has progressively revealed that neuroinflammation is a critical driver in the pathogenesis and progression of various neurological disorders [1, 2, 3]. Within this complex neuroimmune network, astrocytes, the most abundant glial cells in the CNS, have been redefined from their traditional role as mere “supporting cells” to key regulators of neuroinflammation [4, 5, 6]. Beyond maintaining the blood–brain barrier (BBB), regulating synaptic function, and providing metabolic support, astrocytes actively participate in and modulate neuroinflammatory processes through morphological, molecular, and functional remodeling under pathological conditions [7, 8, 9, 10]. From neurodegenerative diseases such as Alzheimer's disease (AD) and Parkinson's disease (PD) to multiple sclerosis (MS), CNS infections, and injury, aberrant astrocyte activation is a nearly ubiquitous feature across diverse neuropathologies [11, 12, 13, 14]. Consequently, a deeper understanding of the multifaceted roles of astrocytes in neuroinflammation is crucial not only for elucidating disease mechanisms but also for identifying novel therapeutic targets.

Advancements in research methodologies, particularly the development of single‐cell transcriptomics, spatial multi‐omics, and epigenetics, are driving a paradigm shift in our comprehension of astrocyte reactivity. Early studies, drawing parallels to the M1/M2 polarization paradigm of macrophages, proposed an A1/A2 dichotomy for astrocytes, aiming to simplify their dual functions in inflammation: wherein the A1 phenotype was considered neurotoxic and the A2 phenotype neuroprotective [15, 16, 17]. Although the A1/A2 classification has provided an accessible framework for describing the opposing inflammatory functions of reactive astrocytes, accumulating single‐cell and single‐nucleus transcriptomic studies indicate that this binary model is insufficient to capture the complexity of astrocyte responses in vivo [18, 19, 20]. Their reactive states constitute a highly heterogeneous and dynamic “continuum,” shaped collectively by disease type, stage, brain region, and cellular crosstalk [9, 21]. This complexity underscores the inadequacy of attempting to categorize astrocyte function based on single markers or simplistic classifications, necessitating a multidimensional, dynamic, and systems‐level perspective to re‐evaluate their role in neuroinflammation.

Across various neurological diseases, astrocytes contribute to and amplify neuroinflammatory processes through multiple mechanisms, which include dysregulation of amyloid‐β (Aβ) plaque metabolism and glutamate homeostasis in AD [22, 23, 24, 25], α‐synuclein‐mediated inflammatory circuits in PD [26, 27, 28, 29], excitotoxicity and metabolic dysfunction in amyotrophic lateral sclerosis (ALS) [30, 31], loss of potassium buffering and lipotoxic responses in Huntington's disease (HD) [32, 33, 34], the A1/A2 phenotypic balance determining repair outcomes following CNS injury [35, 36], and their dual role as innate immune sentinels during infection [37]. Astrocytes thus exhibit highly context‐dependent regulatory behaviors in inflammation across different disease backgrounds. Furthermore, astrocytes do not act in isolation but engage in intricate communication with microglia, neurons, oligodendrocytes, and peripheral immune cells, collectively forming a dynamic glial‐immune network [38, 39, 40, 41, 42]. This interactive system can exacerbate damage or promote repair, with the net output hinging on the specificity, timing, and spatial organization of intercellular signaling pathways.

Against this backdrop, this review aims to systematically summarize the multifaceted mechanisms by which astrocytes contribute to neuroinflammatory responses in neurological diseases and to explore their potential as therapeutic targets. We will begin by examining astrocyte reactivity and phenotypic diversity, then delve into the specific inflammatory mechanisms of astrocytes in AD, PD, ALS, HD, CNS injury, and infection. Subsequently, we will delineate the crosstalk between astrocytes and microglia, neurons, oligodendrocytes, and peripheral immune cells, highlighting their central position within the CNS immune network. Moreover, we will systematically analyze key signaling pathways, epigenetic and metabolic mechanisms regulating astrocytic inflammatory responses, evaluate existing and emerging astrocyte‐targeted therapeutic strategies, and discuss future research directions. Through this systematic synthesis, we hope to provide a comprehensive and in‐depth perspective on the central role of astrocytes in neuroinflammation and to lay a theoretical foundation to further develop next‐generation therapeutics for neurological diseases.

## 2 Astrocyte Reactivity and Neuroinflammatory Regulation

### 2.1 Reactive Transformation of Astrocytes and Molecular Signatures

In response to CNS injury, infection, or neurodegenerative pathology, astrocytes undergo a transition from homeostasis to a reactive state, a process termed “reactive astrogliosis” [43, 44]. This is not a simple “on/off” switch but rather a highly heterogeneous response involving comprehensive morphological, genomic, metabolic, and functional remodeling. Morphologically, reactive astrocytes exhibit hypertrophic cell bodies with thickened and elongated processes, changes often accompanied by the upregulation of intermediate filament proteins, particularly glial fibrillary acidic protein (GFAP). Due to widespread inducibility across diverse CNS injury models, GFAP has become the most classical marker of reactive astrocytes [45, 46]. However, GFAP expression levels vary significantly depending on the injury type, proximity to the lesion, and CNS region, and its upregulation is not a universal feature of all reactive astrocytes. Therefore, GFAP alone cannot fully reveal the functional implications or heterogeneity of reactivity [47].

The molecular alterations in reactivity are more complex and specific. Transcriptomic analyses have unveiled extensive gene expression reprogramming in reactive astrocytes [48, 49, 50]. For instance, under pro‐inflammatory conditions, cells may upregulate complement components (e.g., C1q, C3), pro‐inflammatory cytokines (e.g., IL‐1β, IL‐6, TNF‐α) [51, 52, 53], and chemokines (e.g., CXCL10) [54, 55, 56], while concurrently downregulating a suite of homeostatic molecules, including the glutamate transporter EAAT2/GLT1, neurotrophic factors (e.g., BDNF, GDNF) [57, 58, 59], and extracellular matrix proteins (e.g., SPARCL1, GPC4) [60, 61, 62]. This shift in molecular signature directly corresponds to a functional transition: from normal synaptic support, neurotransmitter clearance, and ion balance maintenance towards diverse, often opposing, functional outputs such as immune regulation, cytotoxicity, and neuroprotection [63, 64]. Consequently, understanding astrocyte reactivity must extend beyond the singular metric of GFAP to encompass its multidimensional, dynamically changing molecular signature landscape.

### 2.2 Phenotypic Diversity of Reactive Astrocytes: From Dichotomy to a Continuum

Early studies, aiming to simplify complexity by analogy to the “M1/M2” polarization paradigm of macrophages, proposed an “A1/A2” dichotomous model for reactive astrocytes [65, 66]. The A1 phenotype is typically induced by the pro‐inflammatory triad of IL‐1α, TNF‐α, and C1q released from activated microglia [65, 67]. Its molecular profile is characterized by the upregulation of pro‐inflammatory mediators, complement cascade components, and neurotoxins, alongside the downregulation of neurotrophic factors and glutamate uptake capacity, demonstrating neurotoxic effects in models of various neurodegenerative diseases such as AD, PD, and MS. Conversely, the A2 phenotype is activated in contexts of tissue repair, such as ischemia, and upregulates neurotrophic factors (e.g., BDNF, VEGF, bFGF) [68], thrombospondins (THBS1/2) [69], and aquaporin‐4, endowing it with neuroprotective and pro‐repair potential. This dichotomy provides a valuable framework to understand the dual roles of astrocytes in inflammation.

However, with the application of high‐throughput technologies like single‐cell RNA sequencing and spatial transcriptomics, it has become increasingly clear that A1 and A2 represent merely two extreme states within a continuum of reactive astrocyte phenotypes [20, 70, 71, 72]. In authentic pathological environments, astrocytes display a far richer diversity of states. For example, distinct disease‐associated astrocyte states, driven by specific signaling pathways (e.g., MAFG/MAT2α in MS) or transcription factors (e.g., unfolded protein response in PD), have been identified across different neurodegenerative diseases. These states are not fixed but exhibit high plasticity, potentially co‐expressing features of both A1 and A2 or dynamically transitioning between states over time. Thus, the current perspective has shifted from a rigid dichotomy towards a more nuanced “continuum” concept, emphasizing that reactive astrocyte phenotypes constitute a continuous, dynamic collection of functional states shaped by specific disease contexts, spatiotemporal dynamics, and intercellular interactions.

### 2.3 Core Regulation of Astrocyte State Transitions

The precise regulation of reactive astrocyte phenotypes relies on a complex network of intracellular and extracellular signals. Extracellular cues serve as the initial drivers of phenotypic induction. The pro‐inflammatory cytokines TNF‐α, IL‐1β, and IFN‐γ, as well as the microglia‐derived triple signal of IL‐1α + TNF‐α + C1q, are key inducers of the A1‐like neurotoxic phenotype [65, 73, 74]. In contrast, anti‐inflammatory factors like transforming growth factor‐β (TGF‐β) tend to promote an A2‐like neuroprotective phenotype. Environmental factors also play significant roles: gut microbiota metabolites can restrict inflammation by activating the aryl hydrocarbon receptor (AHR) to inhibit nuclear factor kappa B (NF‐κB) [75, 76, 77], whereas environmental toxins like the herbicide linuron enhance pro‐inflammatory programs via the SigmaR1‐IRE1α‐XBP1 axis [78, 79].

Intracellularly, the integration and balance of core signaling pathways determine the ultimate cell state. As a master regulator of pro‐inflammatory and neurotoxic gene expression, the NF‐κB pathway plays a dominant role in A1 polarization [80, 81]. The signal transducer and activator of transcription 3 (STAT3) pathway is often associated with tissue repair and the A2 phenotype, yet its function exhibits significant context‐dependency—while STAT3 activation aids in limiting inflammation and promoting repair after spinal cord injury [82, 83], its inhibition may ameliorate pathology in a mouse model of AD [84]. Other critical regulatory nodes include the IRE1α‐XBP1 pathway (involved in endoplasmic reticulum stress and inflammation) [78], the MAFG/MAT2α axis (driving a pathogenic state and suppressing anti‐inflammatory genes in MS) [20], and fluctuations in Ca^2^^+^ signaling (regulates the release of neuroactive substances) [85].

These pathways do not operate in isolation but form an intricate interactive network whose net output determines whether astrocytes exacerbate neural damage or promote repair. Furthermore, interactions between astrocytes and other cells of the CNS (e.g., microglia, neurons, infiltrating immune cells), via cell–cell contact or soluble factors, continuously reshape their own state [24, 86, 87]. Therefore, deciphering the dynamic interplay of these core regulatory mechanisms is crucial for developing therapeutic strategies capable of precisely targeting detrimental astrocyte states while preserving or enhancing their protective functions (Figure 1).

**FIGURE 1:**
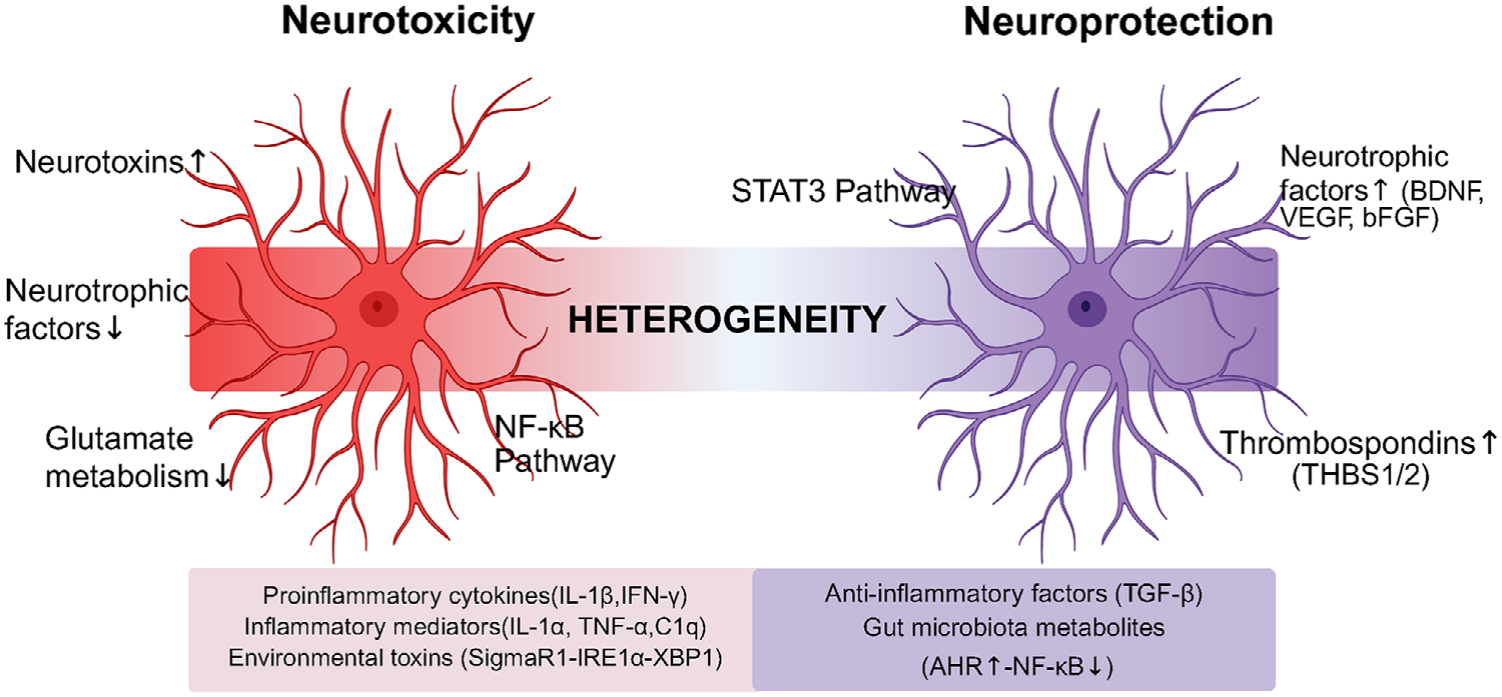
Reactive astrocyte phenotypes along a neurotoxic–neuroprotective continuum. The diagram illustrates the dynamic and heterogeneous nature of astrocyte reactivity, transcending the traditional A1 (neurotoxic) and A2 (neuroprotective) dichotomy. Key molecular drivers and pathways‐such as pro‐inflammatory cytokines (IL‐1β, IFN‐γ) and the NF‐κB pathway promoting neurotoxicity, versus STAT3 activation, thrombospondins (THBS1/2), and neurotrophic factors (BDNF, VEGF, bFGF) supporting neuroprotection—are highlighted. Environmental factors, including gut microbiota metabolites (acting via AHR) and toxins (via SigmaR1‐IRE1α‐XBP1), further shape astrocyte phenotype. This continuum model underscores the context‐dependent functional outputs of reactive astrocytes in neurological diseases. AHR, aryl hydrocarbon receptor; BDNF, brain‐derived neurotrophic factor; bFGF, basic fibroblast growth factor; IFN‐γ, interferon gamma; IL‐1β, interleukin‐1 beta; IRE1α, inositol‐requiring enzyme 1 alpha; NF‐κB, nuclear factor kappa‐B; SigmaR1, sigma non‐opioid intracellular receptor 1; STAT3, signal transducer and activator of transcription 3; THBS1/2, thrombospondin 1/2; VEGF, vascular endothelial growth factor; XBP1, X‐box binding protein 1.

## 3 Mechanisms of Astrocyte‐Mediated Neuroinflammation in Neurological Diseases

### 3.1 AD

AD, the most common neurodegenerative disorder, is pathologically characterized by extracellular Aβ plaque deposition and intracellular neurofibrillary tangles. Astrocytes play a context‐dependent role in AD‐associated neuroinflammation, shifting from early compensatory responses to maladaptive inflammatory and metabolic dysfunction as the disease progresses [88, 89, 90, 91]. In the early stage, astrocytes may contribute to Aβ degradation and clearance by secreting matrix metalloproteinases and other proteolytic mediators [92, 93, 94]. However, persistent Aβ accumulation drives astrocytes toward reactive states characterized by increased GFAP and S100β expression, intermediate filament remodeling, and clustering around Aβ plaques [95]. Compared with other neurodegenerative diseases, astrocyte dysfunction in AD is particularly linked to Aβ metabolism, synaptic impairment, and vascular dysregulation. Reactive astrocytes release inflammatory mediators such as IL‐1β, IL‐6, and TNF‐α, which further activate microglia and maintain a local inflammatory microenvironment [96, 97]. In addition, astrocyte‐expressed molecules, including BACE1 and IFITM3, may promote Aβ production, forming a feed‐forward loop between amyloid pathology and astrocyte reactivity [98]. Astrocytic impairment of EAAT2/GLT‐1 reduces glutamate uptake and contributes to excitotoxic stress, while aberrant intracellular Ca^2^^+^ signaling promotes GABA release through the BEST1 channel, suppressing neuronal activity and impairing synaptic plasticity [99, 100, 101, 102]. Metabolically, reduced astrocyte‐derived lactate weakens neuronal energy support, whereas APOE4 disrupts BBB integrity through the CypA‐NF‐κB‐MMP9 pathway [103, 104]. Therefore, astrocytes in AD are not merely secondary responders to amyloid deposition; rather, they actively connect Aβ pathology, inflammatory amplification, synaptic dysfunction, metabolic insufficiency, and vascular injury.

### 3.2 PD

PD is characterized by the loss of dopaminergic neurons in the substantia nigra and the abnormal aggregation of α‐synuclein. Astrocytes contribute to PD pathogenesis mainly through α‐synuclein‐associated reactivity, inflammatory amplification, metabolic toxicity, and impaired astrocyte–dopaminergic neuron communication [105, 106, 107, 108]. In the early stage of PD, astrocytes can accumulate or internalize α‐synuclein, which promotes reactive remodeling manifested by increased GFAP expression and morphological hypertrophy [109, 110, 111, 112]. These reactive astrocytes release inflammatory mediators such as IL‐1β, TNF‐α, and reactive oxygen species (ROS), thereby strengthening astrocyte–microglia crosstalk and forming a positive feedback loop of neuroinflammation [113]. The astrocytic inflammatory response in PD has several disease‐specific features. Activation of the Janus kinase/signal transducer and activator of transcription 3 (JAK/STAT3) pathway is closely associated with reactive astrogliosis, and its inhibition can reduce neuroinflammation and dopaminergic neuronal damage in experimental models [114, 115]. Astrocytes also support dopaminergic neuron survival through Wnt1/Fzd‐1/β‐catenin signaling, whereas disruption of this pathway weakens neuron–astrocyte protective communication [116]. In addition, astrocytic monoamine oxidase B (MAO‐B) is particularly relevant to PD‐related metabolic toxicity, as it converts MPTP into MPP^+^, directly injuring dopaminergic neurons [117]. Downregulation of NR4A2/Nurr1 in astrocytes further impairs their anti‐inflammatory function, resulting in sustained NF‐κB activation and increased release of neurotoxic factors [118]. These inflammatory mechanisms, combined with glutamate excitotoxicity, impaired K^+^ buffering, and disrupted Ca^2^^+^ homeostasis, collectively accelerate the degeneration of dopaminergic neurons, highlighting the central role of astrocytes in PD pathogenesis.

### 3.3 ALS

ALS is a fatal disease characterized by the progressive loss of motor neurons, in which astrocytes significantly contribute to neuroinflammation and degeneration via non‐cell‐autonomous mechanisms [119, 120, 121]. In ALS, astrocytes expressing mutant SOD1 acquire toxic properties and can directly induce motor neuron degeneration [122, 123]. These cells exhibit downregulated expression or impaired function of the astrocytic glutamate transporter EAAT2/GLT‐1, leading to defective extracellular glutamate clearance and excitotoxic stress on motor neurons [124, 125]. Concurrently, impaired lactate transport in astrocytes weakens their metabolic support for motor neurons, further exacerbating the neuronal energy crisis [122]. The inflammatory response is also prominent in ALS; however, compared with the more generalized inflammatory amplification described in AD and PD, its pathological consequence is closely linked to motor neuron‐selective vulnerability. Astrocytes release pro‐inflammatory factors, including IL‐6, TGF‐β, and TNF‐α, and enhance their neurotoxicity via STAT3‐dependent pathways [126, 127, 128]. Furthermore, changes in the expression of the AMPA receptor subunit GluR2 and aberrant activation of the p75 neurotrophin receptor signaling pathway in astrocytes collectively promote motor neuron apoptosis [129]. In both familial and sporadic ALS models, astrocytes show significant upregulation of inflammation‐related and stress‐response genes, underscoring their contribution to motor neuron degeneration [33, 130, 131]. Notably, transplantation of wild‐type astrocyte precursors can delay disease progression, further confirming the pathogenic role of astrocytes in ALS [132]. These mechanisms collectively reveal that astrocytes drive ALS progression through mutant SOD1‐associated non‐cell‐autonomous toxicity, EAAT2/GLT‐1‐dependent glutamate dysregulation, impaired metabolic support, and STAT3/GluR2/p75‐related enhancement of motor neuron vulnerability.

### 3.4 HD

HD is an inherited neurodegenerative disorder caused by mutant huntingtin (mHTT) protein, with prominent degeneration of striatal neurons. Astrocytes expressing mHTT contribute to HD progression by disrupting potassium and glutamate homeostasis, promoting inflammatory signaling, and undergoing maladaptive metabolic and epigenetic remodeling. One of the most characteristic astrocytic abnormalities in HD is the reduced expression or dysfunction of Kir4.1 potassium channels and EAAT2/GLT‐1. Impaired Kir4.1‐mediated potassium buffering increases extracellular K^+^ levels, while reduced glutamate clearance elevates extracellular glutamate; together, these changes promote neuronal hyperexcitability and Ca^2^^+^ dysregulation in striatal circuits [133, 134]. Abnormal mGluR2/3‐mediated astrocytic Ca^2^^+^ signaling further amplifies neuron‐glia dysfunction [135]. Astrocytes in HD also participate in inflammatory amplification and metabolic stress. In HD models, astrocyte‐mediated NF‐κB signaling is abnormally sustained and can increase pro‐inflammatory cytokine production, supporting a glial contribution to chronic inflammation and disease progression [136, 137]. Metabolically, low glucose can drive striatal astrocytes toward fatty‐acid oxidation, which provides an alternative fuel source but increases ROS and region‐specific vulnerability [138, 139, 140]. Transcriptomic and multi‐omics studies further identify disease‐ and region‐specific astrocyte states with alterations in homeostatic, inflammatory, and lipid‐associated programs [141, 142]. Epigenetic dysregulation may also contribute to HD astrocyte dysfunction: altered chromatin accessibility and transcription have been demonstrated during HD astrocyte differentiation, while broader DNA‐methylation changes are associated with HD and disease progression [143, 144, 145]. These findings position astrocytes as important contributors to HD pathogenesis while underscoring that their states are heterogeneous rather than uniformly neurotoxic.

Although AD, PD, ALS, and HD differ in initiating pathological proteins and vulnerable neuronal populations, astrocyte‐mediated neuroinflammation across these diseases converges on several shared functional modules, including inflammatory cytokine release, impaired glutamate and ion homeostasis, metabolic remodeling, oxidative stress, and reciprocal amplification of microglial and neuronal injury signals. However, the upstream triggers and dominant astrocytic outputs are disease‐context dependent. AD is more closely associated with Aβ metabolism, APOE4‐related vascular dysfunction, and synaptic impairment [88, 89, 90]; PD is characterized by α‐synuclein stress, dopaminergic neuron vulnerability, MAO‐B‐mediated toxicity, and Wnt/STAT3‐related signaling [105, 110, 114, 117]; ALS is dominated by EAAT2/GLT‐1 loss, mutant SOD1‐associated astrocyte toxicity, and motor neuron excitotoxicity [119, 120]; whereas HD is marked by mHTT‐induced disruption of Kir4.1/EAAT2‐mediated ion and glutamate buffering, metabolic remodeling, and disease‐associated astrocyte states [133, 138]. This integrated view helps distinguish common astrocytic inflammatory modules from disease‐specific reactive programs and avoids presenting astrocyte activation as a repetitive generic response.

The mechanisms by which astrocytes participate in neuroinflammation across neurodegenerative diseases are summarized in Figure 2 and Table 1.

**FIGURE 2:**
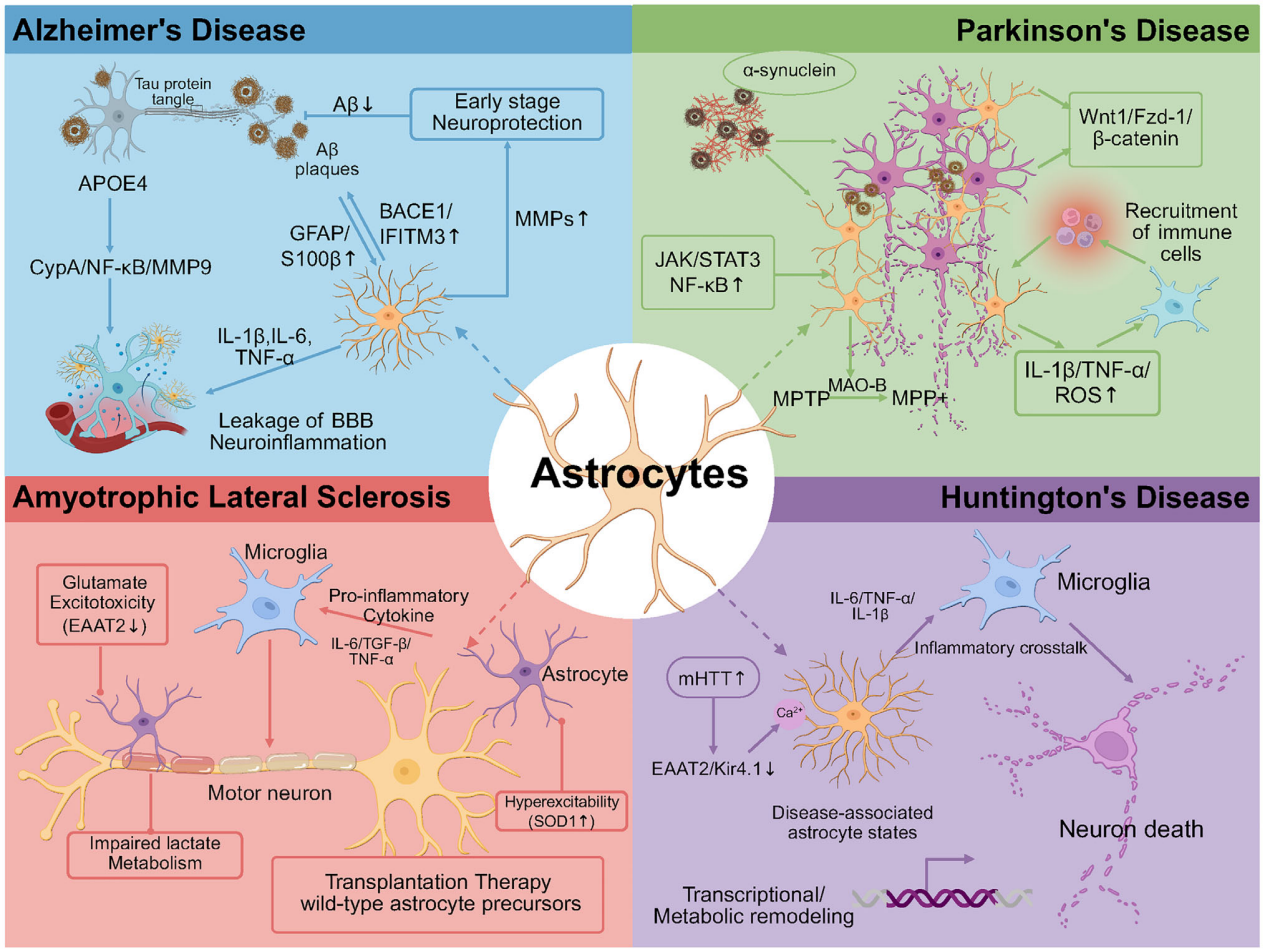
Reactive astrocyte‐mediated pathogenic mechanisms in neurodegenerative diseases. Schematic representation of key astrocytic pathways contributing to AD, PD, ALS, and HD. In AD, astrocytes show Aβ plaque‐associated reactivity, altered GFAP/S100β expression, impaired glutamate homeostasis, and APOE4‐associated blood‐brain barrier dysfunction. In PD, α‐synuclein stress is linked to JAK/STAT3 and NF‐κB signaling, astrocyte‐microglia inflammatory crosstalk, and altered Wnt/β‐catenin support. In ALS, astrocytic EAAT2/GLT‐1 dysfunction, impaired metabolic support, and mutant SOD1‐associated non‐cell‐autonomous toxicity contribute to motor‐neuron vulnerability. In HD, mHTT‐associated loss of Kir4.1/EAAT2 function, inflammatory signaling, and disease‐associated transcriptional and metabolic astrocyte states contribute to striatal circuit dysfunction. The figure emphasizes both shared inflammatory modules and disease‐specific astrocyte programs. BBB, blood‐brain barrier; EAAT2, excitatory amino acid transporter 2; GFAP, glial fibrillary acidic protein; HMGB1, high mobility group box 1; IL, interleukin; mHTT, mutant huntingtin; NF‐κB, nuclear factor kappa‐B; NR4A2, nuclear receptor subfamily 4 group A member 2; RAGE, receptor for advanced glycation end‐products; S100β, S100 calcium‐binding protein β; STAT3, signal transducer and activator of transcription 3; TLR, Toll‐like receptor; TNF‐α, tumor necrosis factor‐alpha; α‐syn, alpha‐synuclein.

**TABLE 1:** Mechanisms of astrocytic involvement in neuroinflammation across neurological diseases.

Diseases	Key Inflammatory Mechanisms	Roles of Astrocytes	Reference
AD	Aβ metabolic dysregulation, plaque‐associated inflammation, impaired glutamate homeostasis, Ca^2^^+^ signaling abnormalities	Initially contributes to Aβ clearance, but persistent Aβ pathology drives maladaptive reactivity, linking inflammation with synaptic and metabolic dysfunction	[92, 93, 94, 96, 97]
PD	α‐synuclein‐associated inflammatory activation, astrocyte–microglia feedback, ROS production, MAO‐B‐mediated metabolic toxicity	Promotes dopaminergic neuron vulnerability through inflammatory circuit amplification and astrocyte‐related metabolic toxicity	[113, 117]
ALS	Mutant SOD1‐related astrocyte toxicity, EAAT2/GLT‐1 dysfunction, impaired glutamate clearance, STAT3‐related inflammatory activation	Drives motor neuron‐selective excitotoxicity and weakens astrocyte‐dependent metabolic support	[122, 123, 126, 127, 128]
HD	mHTT‐associated astrocyte dysfunction, Kir4.1/EAAT2 impairment, extracellular K^+^ and glutamate imbalance	Promotes striatal neuronal hyperexcitability and contributes to glutamate/K^+^‐mediated neuronal dysfunction	[133, 134]
CNS Injury	Temporal transition among inflammatory, scar‐forming, and repair‐associated reactive states, DAMPs activating TLR/purinergic receptors, chemokine release	Pro‐inflammatory in acute phase, pro‐repair in subacute phase, sustains inflammation in chronic phase	[146, 147, 148, 149, 150, 151, 152, 153]
CNS Infection	PRR recognition of PAMPs, IFN‐I antiviral response, MMP‐mediated BBB disruption	Acts as sentinels of innate immunity; exhibits dual roles in defense and immunopathology	[160, 161]
Other Diseases (Epilepsy, MS, ASD)	Chemokine release, complement activation, aberrant synaptic pruning, unfolded protein response/NF‐κB pathway activation	Regulates immune cell recruitment, modulates synaptic function and behavioral outcomes	[171, 172, 173, 174, 178, 179, 180, 181, 182, 183, 184]

### 3.5 CNS Injury‐Related Disorders

In CNS injury‐related disorders, astrocytes dynamically regulate the balance between inflammation and repair through a spectrum of injury‐associated reactive states rather than through a simple transition between two fixed phenotypes (Figure 3). Although many early studies described neurotoxic “A1” and neuroprotective “A2” astrocytes, these terms should be interpreted as simplified labels for particular molecular and functional programs within a broader continuum of astrocyte reactivity. During the acute phase (24–72 h) of traumatic brain injury (TBI), astrocytes are activated by mechanical stress, BBB disruption, and damage‐associated molecular patterns (DAMPs) like HMGB1 and ATP. Signaling through Toll‐like receptor (TLR) and purinergic receptors triggers Ca^2^^+^ influx, activating NF‐κB, STAT3, and mitogen‐activated protein kinase (MAPK) pathways. This leads to cellular hypertrophy, upregulated GFAP expression, and the release of pro‐inflammatory cytokines (IL‐6, TNF‐α, IL‐1β) and chemokines (CCL2, CXCL10), which recruit neutrophils, monocytes, and T cells, amplifying neuroinflammation [146, 147, 148, 149]. These early inflammatory programs resemble previously described A1‐like features, including increased C3, IL‐6, and TNF‐α expression, but they should be viewed as part of a temporally evolving injury response rather than as a stable astrocyte subtype. In the subacute phase, astrocytes upregulate the anti‐inflammatory cytokine IL‐10 and neuroprotective molecules like IGF‐1 via STAT3 and TGF‐β signaling, and secrete CSPG and laminin, containing inflammation and promoting angiogenesis [150, 151]. These responses overlap with earlier descriptions of A2‐like activity, but they also coexist with inflammatory and scar‐forming functions, indicating that astrocytes often occupy mixed or transitional states. During the chronic phase, astrocytes maintain pro‐inflammatory markers like C3d, and signaling via DAMPs‐RIPK3 contributes to persistent inflammation and neurodegeneration [152, 153]. Epigenetic regulation (DNA methylation) and abnormal Cx43 hemichannel opening further influence phenotypic stability [154, 155]. In ischemic stroke (cerebral infarction), astrocytes are similarly activated by ischemia, releasing CCL20 to recruit dendritic cells and lymphocytes. Dysregulated sphingolipid metabolism leads to lactosylceramide accumulation, which activates the cPLA2‐mitochondrial antiviral signaling (MAVS)‐NF‐κB signaling pathway, promoting a pro‐inflammatory transcriptional program [156, 157, 158, 159]. Concurrently, MAVS activation reduces lactate production, impairing astrocyte‐neuron metabolic coupling and exacerbating neurodegeneration. Therefore, post‐injury outcomes are determined by the spatiotemporal coordination of multiple reactive astrocyte states.

**FIGURE 3:**
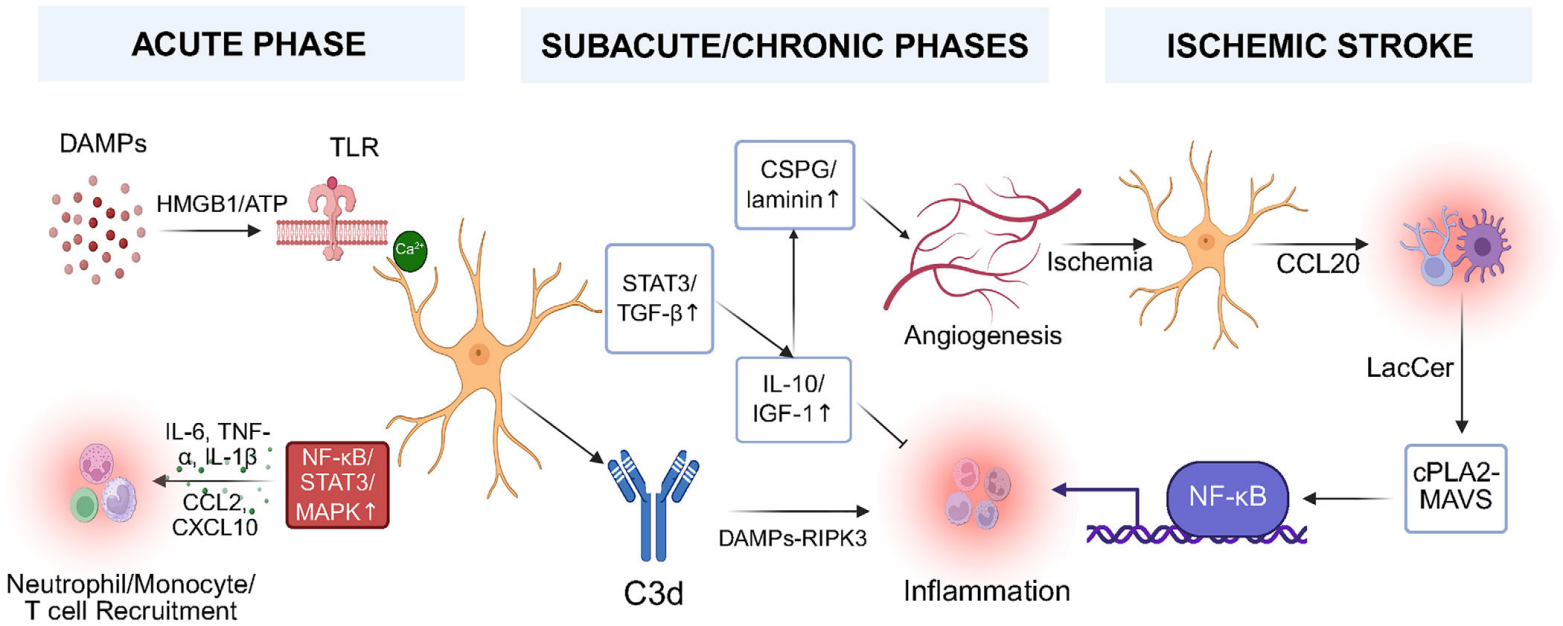
Temporal dynamics of astrocyte‐mediated inflammation and repair following central nervous system injury. This figure outlines phase‐dependent astrocyte responses after CNS injury. In the acute phase, DAMPs such as HMGB1 and ATP activate TLR/purinergic signaling, NF‐κB/STAT3/MAPK pathways, and inflammatory cytokine/chemokine release. During subacute and chronic phases, repair‐associated, inflammatory, and scar‐forming programs may coexist, including IL‐10/IGF‐1 signaling, extracellular‐matrix remodeling, angiogenesis, and persistent C3d‐associated inflammation. In ischemic stroke, CCL20‐mediated immune‐cell recruitment and a distinct lactosylceramide (LacCer)‐cPLA2‐MAVS‐NF‐κB axis represent parallel inflammatory mechanisms rather than a single linear pathway. ATP, adenosine triphosphate; cPLA2, cytosolic phospholipase A2; C3d, complement C3d; CCL20, C‐C motif chemokine ligand 20; CNS, central nervous system; DAMPs, damage‐associated molecular patterns; HMGB1, high mobility group box 1; IGF‐1, insulin‐like growth factor 1; IL, interleukin; LacCer, lactosylceramide; MAPK, mitogen‐activated protein kinase; MAVS, mitochondrial antiviral signaling protein; NF‐κB, nuclear factor kappa‐B; STAT3, signal transducer and activator of transcription 3; TLR, toll‐like receptor.

### 3.6 Infectious Diseases of the CNS

In CNS infectious diseases, astrocytes act as core components of innate immunity, recognizing pathogen‐associated molecular patterns via pattern recognition receptors to initiate anti‐pathogen responses (Figure 4). In viral encephalitis, astrocytes express Toll‐like receptors (TLR3), RIG‐I, and MDA5, recognizing viral double‐stranded RNA. This activates the MAVS and NF‐κB pathways, inducing the production of type I interferons (IFN‐I) [160, 161]. IFN‐I drives the expression of interferon‐stimulated genes like RNase L, which directly cleaves viral RNA, restricting replication [162]. However, viruses like Zika virus (ZIKV) may exploit negative feedback mechanisms, inducing aryl hydrocarbon receptor (AHR) expression to suppress IFN‐I production and evade immune clearance [163, 164]. Astrocyte heterogeneity influences antiviral efficacy, with specific subpopulations highly expressing interferon‐stimulated genes, enhancing defense capabilities [165]. In tuberculous meningitis (TBM), astrocytes recognize *Mycobacterium tuberculosi*s components via TLRs, activating NF‐κB and MAPK signaling and releasing IL‐6, TNF‐α, and chemokines like CCL2, which recruit monocytes and T cells to the infection site. Astrocytes may also upregulate major histocompatibility complex class II (MHC‐II) and co‐stimulatory molecules, potentially participating in T cell activation, although their antigen‐presenting function remains debated [166, 167, 168]. Furthermore, astrocyte‐derived matrix metalloproteinases disrupt the BBB, facilitating immune cell infiltration and exacerbating tissue damage while attempting to control infection [169, 170]. These responses highlight the dual nature of astrocytes in infectious diseases: they can coordinate immune defense but may also mediate immunopathology.

**FIGURE 4:**
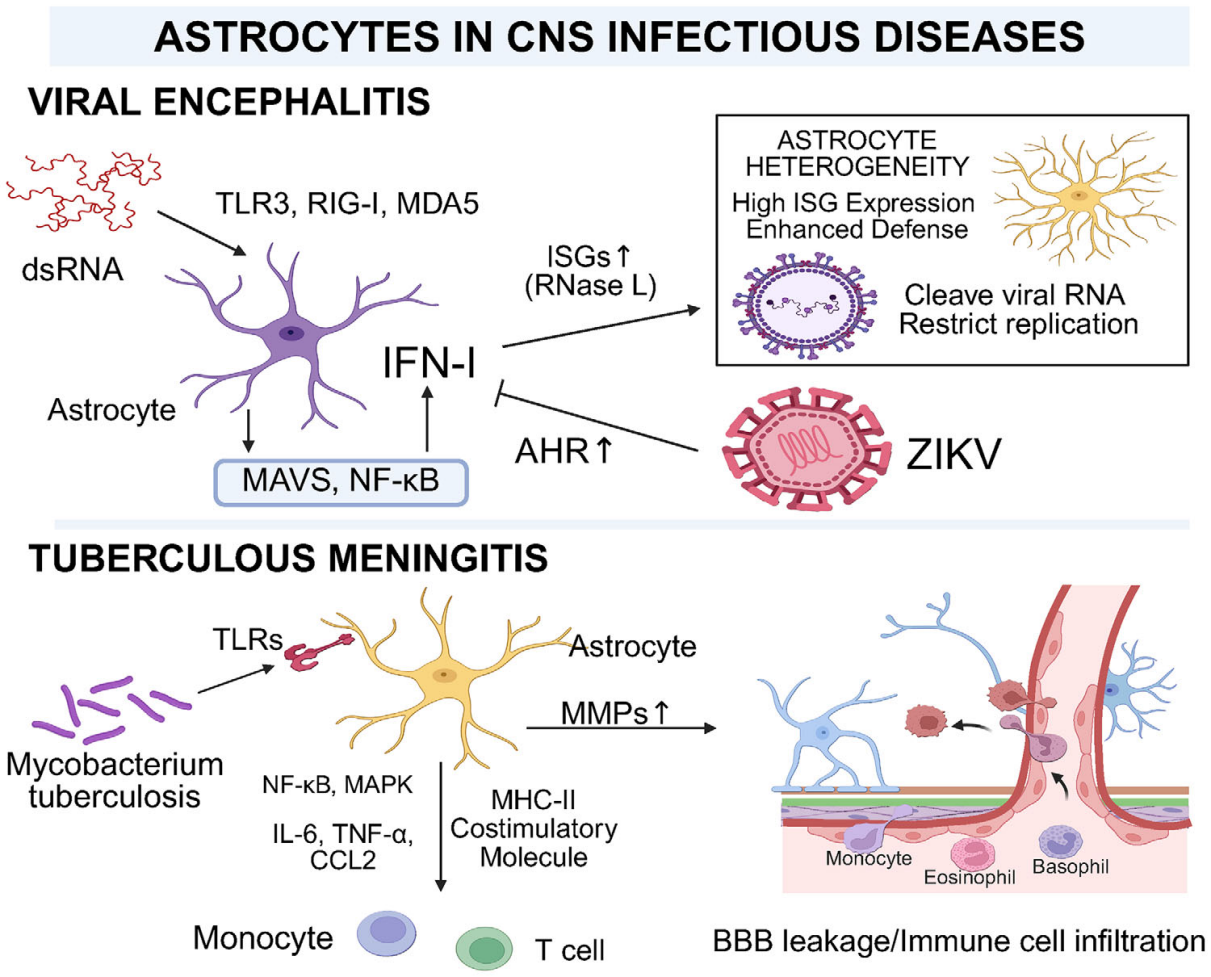
Dual roles of astrocytes in central nervous system infectious diseases. The diagram depicts astrocyte responses in viral encephalitis and tuberculous meningitis (TBM). In viral infection, astrocytes sense viral dsRNA via pattern recognition receptors (TLR3, RIG‐I, MDA5), activating MAVS and NF‐κB pathways to induce type I interferons (IFN‐I) and interferon‐stimulated genes (ISGs, e.g., RNase L) for antiviral defense. However, viruses like ZIKV may exploit AHR upregulation to suppress IFN‐I production. Astrocyte heterogeneity influences defense efficacy. In bacterial infection (TBM), TLR recognition of *M. tuberculosis* components triggers NF‐κB/MAPK signaling, pro‐inflammatory mediator release, immune cell recruitment, and potential antigen presentation (MHC‐II), alongside MMP‐mediated blood–brain barrier disruption, illustrating their dual protective and pathological roles. AHR, aryl hydrocarbon receptor; dsRNA, double‐stranded RNA; IFN‐I, type I interferon; ISGs, interferon‐stimulated genes; MAPK, mitogen‐activated protein kinase; MAVS, mitochondrial antiviral‐signaling protein; MDA5, melanoma differentiation‐associated protein 5; MHC‐II, major histocompatibility complex class II; MMP, matrix metalloproteinase; NF‐κB, nuclear factor kappa‐B; RIG‐I, retinoic acid‐inducible gene I; RNase L, ribonuclease L; TBM, tuberculous meningitis; TLR3, toll‐like receptor 3; ZIKV, Zika virus.

### 3.7 Other Neurological Disorders

Astrocytes profoundly influence the progression of other neurological disorders through inflammatory mechanisms. In MS, astrocyte heterogeneity is significant: in relapsing‐remitting MS, the depletion of reactive astrocytes increases disease severity, suggesting a protective role; whereas in secondary progressive MS, astrocytes promote inflammation and neurodegeneration [171, 172, 173, 174]. Specific subsets, such as C3+ neurotoxic astrocytes and LAMP1+TRAIL+ anti‐inflammatory astrocytes, balance disease progression [20, 73]. Astrocytes produce chemokines like CCL2, CXCL10, and CXCL12, recruiting monocytes and T cells into the CNS. They interact with microglia via signaling protein 4D‐plexinB1/2 and ephrinB3–EPHB3, amplifying inflammation [175, 176]. T cell‐derived granulocyte‐macrophage colony‐stimulating factor (GM‐CSF) drives the differentiation of pathogenic astrocytes via MAFG–MAT2α signaling, downregulating the NRF2 antioxidant response, while DNA methylation stabilizes the pro‐inflammatory phenotype [177]. Environmental factors like herbicides activate astrocyte pathogenicity via the SIGMAR1–IRE1α–XBP1 unfolded protein response pathway, whereas microbial metabolites like tryptophan derivatives exert anti‐inflammatory effects via AHR‐mediated inhibition of NF‐κB signaling [24, 78]. In epilepsy, astrocyte dysfunction, such as EAAT2 downregulation, leads to glutamate accumulation and excitotoxicity. Abnormal Ca^2^^+^ signaling and dysregulated GABA release promote seizure activity [178, 179, 180, 181, 182]. Inflammatory cytokines IL‐1β and TNF‐α activate the NF‐κB pathway, exacerbating synaptic abnormalities and neuronal hyperexcitability. In autism spectrum disorder, astrocytes influence neural circuit development through complement cascade‐ and IL‐33‐mediated aberrant synaptic pruning [183, 184]. Ca^2^^+^ signaling controls GABAergic transmission, modulating anxiety and repetitive behaviors [185, 186], while pro‐inflammatory signals like the unfolded protein response and NF‐κB may drive behavioral deficits [187, 188, 189]. These mechanisms collectively elucidate the central inflammatory role of astrocytes across neurological diseases, providing new perspectives for targeted therapies (Table 1).

## 4 Astrocyte Crosstalk with Other Cells

### 4.1 Astrocyte–Microglia Crosstalk

Bidirectional communication between astrocytes and microglia represents a central mechanism for regulating neuroinflammation in the central nervous system [20, 190, 191]. Under physiological conditions, they work in concert to maintain the homeostasis of the neuronal microenvironment; whereas under pathological conditions, their interactions can significantly exacerbate or ameliorate neuroinflammation and neurodegeneration. Studies show that microglia, upon receiving inflammatory stimuli, such as lipopolysaccharide, can release factors such as IL‐1α, TNF‐α, and C1q, inducing the transformation of astrocytes into a neurotoxic phenotype [65, 67]. This phenotype is characterized by impaired phagocytic capacity, reduced neurotrophic factor expression, and the secretion of undefined neurotoxic substances‐a process observed in various neurodegenerative diseases, including HD, AD, and MS [192, 193]. Further mechanistic insights reveal that the AHR signaling pathway in microglia modulates astrocytic pro‐inflammatory gene expression by regulating the production of VEGF‐B and TGF‐α, which act through FLT‐1 and ErbB1 receptors, respectively, thereby exerting dual pro‐ or anti‐inflammatory effects in disease models like experimental autoimmune encephalomyelitis [191, 194]. Furthermore, the SDF1α‐CXCR4 signaling pathway, in the presence of TNF‐α, can elevate intracellular calcium levels in astrocytes and trigger glutamate release, leading to neuronal excitotoxicity, further illustrating how microglia regulate astrocyte inflammatory responses and neurotoxic potential via cytokine networks [191]. Notably, astrocytes can also reciprocally regulate microglial function, for instance, by secreting factors like GM‐CSF and IL‐6 to activate microglia [195, 196, 197], or providing feedback inhibition of their pro‐inflammatory activity via IL‐10/TGF‐β signaling [198]. Recent research has also identified the SigmaR1–IRE1α–XBP1 signaling axis in astrocytes as a regulator of their crosstalk with microglia, influencing neuroinflammatory progression [78]. These complex bidirectional dialogues not only underscore the central role of the glial network in disease pathogenesis but also provide novel molecular foundations and therapeutic avenues for targeted interventions.

### 4.2 Astrocyte–Oligodendrocyte Interactions

The interactions between astrocytes and oligodendrocytes play a pivotal role in myelin maintenance, inflammatory responses, and neural repair. Traditionally viewed as passive targets of immune responses, recent studies indicate that oligodendrocytes actively participate in immune regulation and express various cytokine receptors, engaging in bidirectional communication with astrocytes [199, 200]. In the context of neuroinflammation, activated astrocytes release mediators such as TNF, Fas ligand, and glutamate, which induce oligodendrocyte apoptosis, thereby inhibiting remyelination and exacerbating neuronal death. This mechanism is implicated in the pathogenesis of autoimmune diseases like MS [201, 202]. Conversely, astrocytes can also recruit oligodendrocyte precursor cells to injury sites by secreting chemokines such as CXCL1, IL‐8, and CCL2, and promote their differentiation into mature myelinating cells via signals such as ciliary neurotrophic factor, thereby enhancing remyelination and functional recovery [199, 203]. In addition, oligodendrocytes themselves can secrete pro‐inflammatory factors including IL‐1β, CCL2, IL‐17, and IL‐6, activating the NF‐κB signaling pathway in astrocytes and forming a positive feedback loop that amplifies CNS inflammation [72, 204]. Intriguingly, oligodendrocytes have been found to disrupt BBB by competitively occupying perivascular spaces, affecting the interactions between astrocytic and endothelial cells, and further promoting immune cell infiltration and disease progression [205]. These findings not only expand our understanding of the immunomodulatory functions of oligodendrocytes but also highlight the complexity and plasticity of subtype‐specific astrocyte‐oligodendrocyte interactions in neuroinflammation and repair, offering new perspectives for understanding demyelinating disease mechanisms and developing pro‐remyelination strategies.

### 4.3 Astrocyte–Neuron Interactions

The interactions between astrocytes and neurons extend far beyond early concepts of mere structural support, playing critical roles in synaptogenesis, neurotransmitter balance, metabolic support, and inflammation regulation. Under healthy conditions, astrocytes participate in regulating synaptic transmission by secreting synaptogenic factors to promote synaptogenesis [206], expressing glutamate transporters (e.g., GLAST, GLT‐1) to clear synaptic glutamate and prevent excitotoxicity [207, 208], and providing metabolic support to neurons via the lactate shuttle mechanism [209]. However, under neuroinflammatory conditions, astrocyte function is often significantly altered, characterized by NF‐κB pathway activation leading to excessive nitric oxide production, decreased glutamate uptake capacity, and reduced synthesis of neurotrophic factors, consequently inducing neuronal death [78, 210, 211]. For instance, in HD models, reduced astrocytic Kir4.1 expression or function elevates extracellular potassium and contributes to striatal neuronal hyperexcitability [212]. Furthermore, metabolic remodeling in astrocytes affects their energy supply to neurons; for example, lactosylceramide activation of the cPLA2‐MAVS pathway disrupts the hexokinase 2‐MAVS interaction, activating NF‐κB‐driven pro‐inflammatory responses while simultaneously inhibiting glycolysis and lactate production, thereby weakening metabolic support for neurons [53]. Notably, astrocytes can also exert neuroprotective effects under specific conditions, such as promoting dopaminergic neuron survival and regeneration via Wnt1 signaling, or expressing molecules like CSPG4/5 to support axonal growth [213, 214]. These discoveries not only reveal the dual role of astrocytes in neurodegenerative diseases but also emphasize their central position in neuron‐glia metabolic coupling and inflammatory regulation, providing new targets for developing neuroprotective therapies.

### 4.4 Astrocyte‐Mediated Recruitment of Peripheral Immune Cells

As integral components of the BBB, astrocytes act as gatekeepers in the interaction between the CNS and the peripheral immune system. During neuroinflammation, astrocytes actively regulate the recruitment and infiltration of peripheral immune cells (including T cells, monocytes, B cells, etc.) into the CNS by secreting various chemokines (e.g., CCL2, CXCL10, CXCL12) [215, 216]. Among these, CCL2 and CXCL10, as key downstream chemokines of the NF‐κB pathway, promote the migration of monocytes and T cells into perivascular spaces and the parenchyma in models of experimental autoimmune encephalomyelitis and MS, significantly influencing disease severity [217]. In addition, astrocytes can exert anti‐inflammatory effects by expressing FasL and TRAIL to induce apoptosis in infiltrating lymphocytes [73, 218]; conversely, their production of B‐cell activating factor supports B cell survival and antibody production, participating in the regulation of humoral immune responses [219]. Notably, the interaction between astrocytes and peripheral immune cells is bidirectional and temporally dynamic: on one hand, IFN‐γ derived from Th1 cells can induce the expression of MHC‐II and co‐stimulatory molecules on astrocytes, enhancing their antigen‐presenting capacity [220, 221]; IL‐17 and GM‐CSF secreted by Th17 cells further activate pro‐inflammatory programs in astrocytes, forming a positive feedback loop that amplifies the inflammatory response [222, 223]. On the other hand, regulatory T cells suppress astrocyte pathogenicity by secreting molecules like IL‐10, promoting inflammation resolution and tissue repair [224, 225]. Recent research also identified that meningeal NK cells induce TRAIL expression on astrocytes via IFN‐γ, which subsequently clears pathogenic T cells, uncovering a novel gut‐brain axis pathway regulating central inflammation [226]. These complex interactive networks not only deepen our understanding of neuroimmune interface functionality but also provide new strategies and targets for treating neuroinflammatory diseases by modulating the dialogue between astrocytes and immune cells (Figure 5).

**FIGURE 5:**
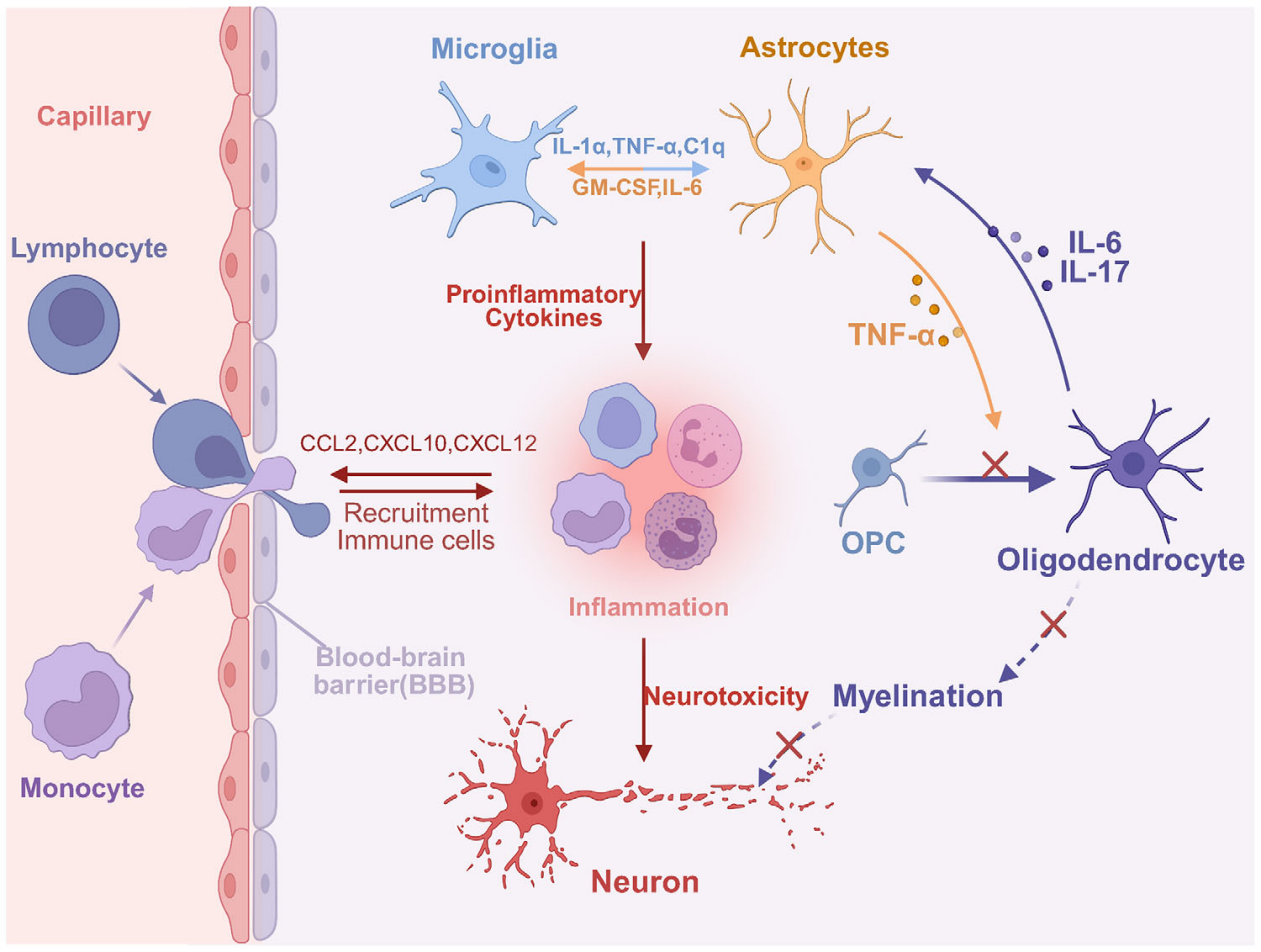
Astrocyte‐centric intercellular communication network in neuroinflammation. This schematic highlights the pivotal role of astrocytes in coordinating neuroimmune responses through bidirectional communication with other CNS cells and peripheral immune cells. Key interactions include: Microglia‐astrocyte crosstalk, where microglial IL‐1α, TNF‐α, and C1q can induce neurotoxic astrocytes, which in turn release GM‐CSF and IL‐6 to feedback on microglia; Astrocyte–oligodendrocyte interactions, involving both detrimental (TNF‐α‐induced apoptosis) and supportive (CXCL1‐mediated OPC recruitment) signals; Recruitment of peripheral monocytes and lymphocytes via astrocyte‐derived chemokines (CCL2, CXCL10, CXCL12) across the BBB. The figure positions the astrocyte as a central regulator of the inflammatory milieu. BBB, blood–brain barrier; C1q, complement C1q; CCL2, C‐C motif chemokine ligand 2; CNS, central nervous system; CXCL1, C‐X‐C motif chemokine ligand 1; CXCL10, C‐X‐C motif chemokine ligand 10; CXCL12, C‐X‐C motif chemokine ligand 12; GM‐CSF, granulocyte‐macrophage colony‐stimulating factor; IL‐1α, interleukin‐1 alpha; IL‐6, interleukin‐6; OPC, oligodendrocyte precursor cell; TNF‐α, Tumor necrosis factor‐alpha.

## 5 Molecular Mechanisms Regulating Astrocyte Inflammatory Responses

### 5.1 Key Signaling Pathways

The initiation and fine‐tuning of astrocyte inflammatory responses rely on a complex intracellular signaling network, wherein several highly synergistic and counterbalancing pathways play central roles (Figure 6). The JAK/STAT3 pathway is recognized as a central route for initiating reactive astrogliosis [24, 227, 228]. Cytokines such as IL‐6 and ciliary neurotrophic factor (CNTF) activate JAK kinases via the gp130 receptor [228], leading to STAT3 phosphorylation, dimerization, and nuclear translocation, which directly drives the transcription of a series of reactivity‐associated genes, including GFAP and vimentin. Sustained STAT3 activation has been observed in animal models of various neurodegenerative diseases, including AD, HD [84, 229], and MS [230], underscoring its central role [229]. However, STAT3 function exhibits significant context‐dependency: astrocyte‐specific STAT3 deletion in spinal cord injury impairs motor function recovery and exacerbates inflammation [231, 232, 233], whereas STAT3 inhibition in AD models reduces Aβ plaques and improves cognitive function [84], revealing its dual role in pro‐inflammatory and repair processes.

**FIGURE 6:**
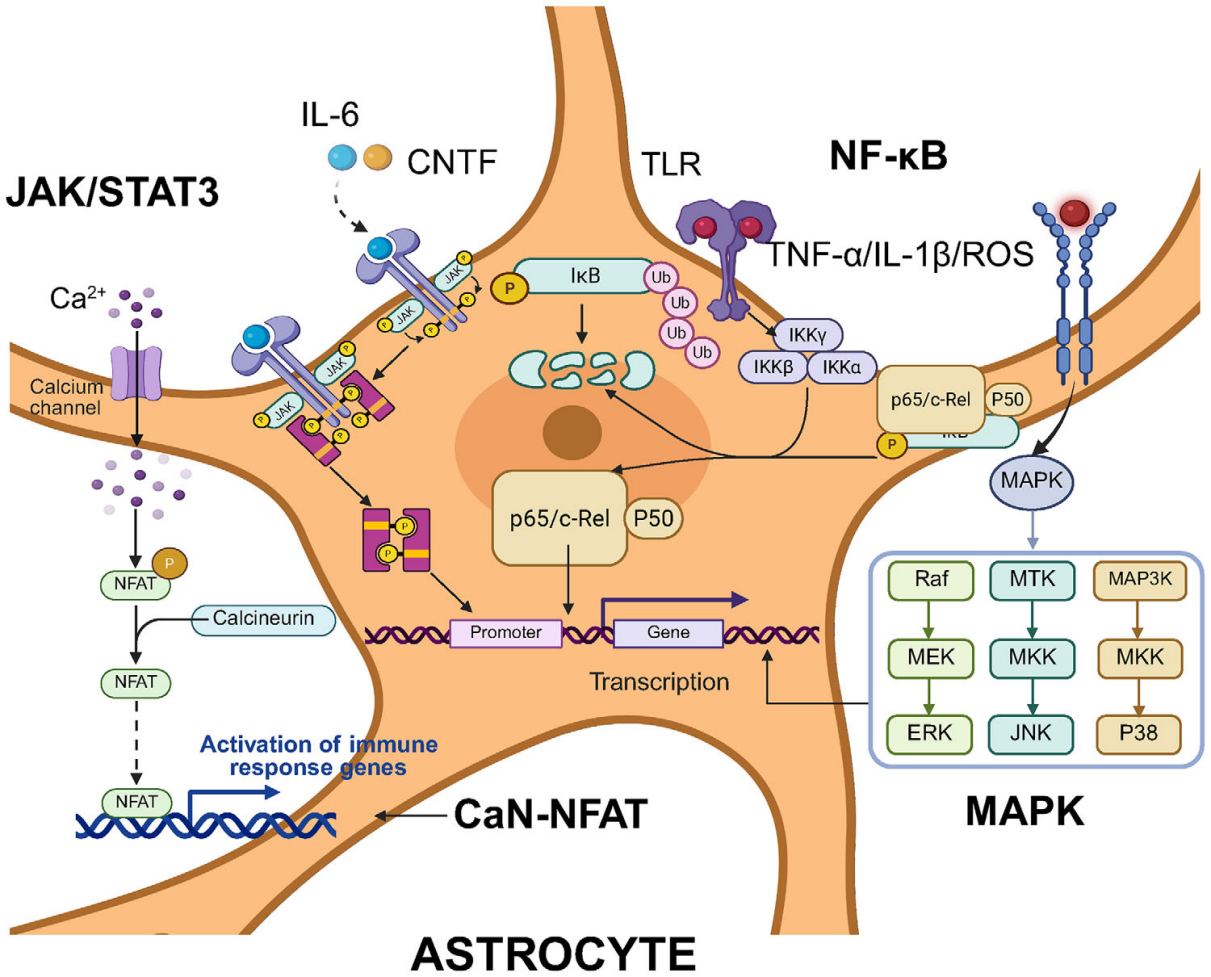
Core intracellular signaling pathways governing astrocyte inflammatory responses. The diagram summarizes major signal transduction pathways implicated in astrocyte reactivity and neuroinflammation. Key pathways include: the JAK/STAT3 pathway, activated by cytokines like IL‐6 and CNTF, driving reactive astrogliosis; the NF‐κB pathway, activated by TNF‐α, IL‐1β, TLR ligands, or ROS, leading to pro‐inflammatory gene transcription; the MAPK cascade (ERK, JNK, p38), coordinating proliferation, migration, and inflammation; and the Ca^2^^+^‐calcineurin‐NFAT pathway, linking calcium fluctuations to inflammatory gene expression. Crosstalk between these pathways and negative feedback mechanisms ensure context‐dependent and finely tuned astrocyte responses to diverse stimuli. Ca^2^^+^, calcium ion; CNTF, ciliary neurotrophic factor; ERK, extracellular signal‐regulated kinases; IL‐1β, interleukin‐1 beta; IL‐6, interleukin‐6; JAK, Janus kinase; JNK, c‐Jun N‐terminal kinases; MAPK, mitogen‐activated protein kinase; NF‐κB, nuclear factor kappa‐B; NFAT, nuclear factor of activated T‐cells; p38, p38 mitogen‐activated protein kinases; ROS, reactive oxygen species; STAT3, signal transducer and activator of transcription 3; TLR, toll‐like receptor; TNF‐α, tumor necrosis factor‐alpha.

The NF‐κB pathway is another key pro‐inflammatory signaling axis, activated by diverse stimuli including TNF‐α, IL‐1β [78, 234], LPS, and ROS [235]. Activated NF‐κB drives the expression of numerous pro‐inflammatory cytokines, chemokines, and complement components, and is a key driver of the A1‐like neurotoxic astrocyte phenotype [65]. Notably, the NF‐κB pathway appears to modulate rather than initiate reactivity; for instance, its inhibition in an ALS model had minimal effect on the number of GFAP‐positive cells [236]. Furthermore, the MAPK pathways (including ERK, JNK, p38) respond to growth factors (e.g., EGF, FGF) and inflammatory signals, coordinating astrocyte proliferation, migration, and pro‐inflammatory mediator production [237, 238, 239, 240]. The calcineurin‐NFAT pathway is activated upon increased intracellular Ca^2^^+^ levels [241] and participates in regulating astrocyte inflammatory responses; its hyperactivation in AD and PD models [242] is closely associated with pathological progression. Crucially, these pathways do not operate in isolation but form an intricate network with extensive crosstalk. For example, STAT3 and NF‐κB can physically interact to co‐regulate target genes; deubiquitinating enzymes A20 and OTUB1 finely tune NF‐κB activity via negative feedback mechanisms, preventing excessive inflammation amplification [243]. Microbial metabolites can inhibit NF‐κB by inducing SOCS2 [77, 244], illustrating the connection between environment and cellular signaling. This complex signal integration network ensures that astrocytes can interpret the myriad cues in the microenvironment and output highly heterogeneous reactive phenotypes ranging from neurotoxic to neuroprotective.

### 5.2 Epigenetic Regulatory Mechanisms

Astrocyte inflammatory responses are not only rapidly activated by transient signals but are also profoundly regulated by epigenetic mechanisms, which provide a form of “molecular memory,” enabling differentiated responses to prior stimuli, i.e., forming “trained immunity” or “immune tolerance” [245, 246, 247]. Histone modifications represent a key class of such mechanisms. Following stimuli like cerebral ischemia, levels of the transcription‐activating mark H3K4me3 are increased in astrocytes, while levels of the repressive mark H3K9me3 are decreased, overall promoting the transcription of pro‐angiogenic genes like VEGF, impacting long‐term pathological changes [248]. Histone deacetylases (HDACs) play complex roles [249, 250, 251]: repeated LPS stimulation can induce HDAC6‐mediated histone deacetylation, leading to reduced production of pro‐inflammatory factors like IL‐6, establishing a tolerant state. More importantly, intercellular crosstalk can induce profound epigenetic remodeling. Microglia‐astrocyte interactions can activate the nuclear receptor Nurr1 [118], which recruits the co‐repressor complex CoREST (containing HDAC1) to the promoter regions of pro‐inflammatory genes, leading to long‐term suppression of NF‐κB‐driven neurotoxic responses via deacetylation, constituting a critical *trans*‐repression circuit.

DNA methylation is another core epigenetic mechanism, involving the addition of methyl groups to DNA to repress gene expression [252]. In MS and its model experimental autoimmune encephalomyelitis (EAE), studies have identified a pathogenic astrocyte subset driven by GM‐CSF from pro‐inflammatory T cells [253]. This subset is characterized by upregulation of the MAFG/MAT2α signaling axis. The MAFG homodimer cooperates with MAT2α (involved in S‐adenosylmethionine synthesis) to competitively inhibit the activity of the neuroprotective transcription factor NRF2, while simultaneously promoting DNA methylation at key genomic regions, including NRF2 response elements, thereby long‐term “locking” the expression of antioxidant and anti‐inflammatory genes and stabilizing astrocytes in a pro‐inflammatory state [254]. Experiments confirmed that specific knockout of MAFG/MAT2α in astrocytes significantly ameliorates EAE, strongly demonstrating the pathogenic importance of this epigenetic module [255]. These findings collectively indicate that epigenetic reprogramming guided by intercellular interactions and chronic inflammatory signals is a core mechanism shaping and long‐term stabilizing astrocyte reactive states. This “memory” capability explains why neuroinflammation can persist or recur after the initial stimulus has subsided, and provides novel perspectives and targets for understanding the chronicity of neurological diseases and developing therapies that can reset epigenetic states for durable efficacy.

### 5.3 Metabolic Reprogramming

Astrocyte inflammatory responses are accompanied by profound metabolic reprogramming, which not only provides energy and biosynthetic precursors for their own activation but also deeply influences the inflammatory process and neuronal survival through the direct regulatory roles of metabolites. Under neuroinflammatory conditions, pro‐inflammatory signals can drive astrocytes to shift from traditional oxidative phosphorylation towards glycolysis. This metabolic switch, reminiscent of the “Warburg effect,” rapidly generates ATP and synthesizes macromolecules to meet the bioenergetic demands of cell proliferation and massive secretion of inflammatory mediators [256, 257, 258, 259]. Lactate metabolism is a hub of astrocyte‐neuron metabolic coupling [156, 260, 261]. Under homeostasis, astrocytes produce lactate via glycolysis and shuttle it to neurons via monocarboxylate transporters, serving as a crucial energy substrate vital for maintaining synaptic function and neuronal survival. However, in the reactive state, this key supportive function is often impaired. For instance, specific pro‐inflammatory signaling pathways (e.g., cPLA2‐MAVS axis) have been shown to inhibit hexokinase‐2‐mediated lactate production in astrocytes, thereby depriving neurons of their energy source and exacerbating excitotoxicity and neurodegeneration [191, 262, 263]. This loss of metabolic support has been repeatedly documented in experimental models of various neurodegenerative diseases, including AD, PD, HD, and ALS [31, 180, 264, 265].

Furthermore, the production of ROS serves both as an inflammatory mediator and a consequence of metabolic dysfunction [266, 267, 268]. Excessive ROS can activate redox‐sensitive pro‐inflammatory pathways such as NF‐κB and promote cellular damage and DAMP release [269, 270, 271]. Glutamate uptake and metabolism by astrocytes is one of their core homeostatic functions, primarily executed by the high‐affinity transporter EAAT2 (GLT1) [272, 273, 274]. In the reactive state, EAAT2 expression and function are often downregulated, leading to impaired synaptic glutamate clearance, triggering neuronal hyperexcitability and Ca^2^^+^ influx‐mediated excitotoxicity‐a central pathological process common to many neurodegenerative diseases. Concurrently, the pro‐inflammatory cytokine TNF‐α can further promote glutamate release from astrocytes themselves, creating a vicious cycle that continuously damages neurons [275, 276].

Circadian rhythm regulation provides an additional layer linking astrocyte metabolism to neuroinflammation, oxidative stress, and glutamate homeostasis. Circadian disruption has been increasingly implicated in neurodegenerative disorders, including PD, and may interact with inflammatory signaling, mitochondrial dysfunction, and redox imbalance [277, 278, 279]. In this context, the excitatory amino acid transporter family is particularly relevant because astrocytic EAATs maintain glutamate homeostasis in the synaptic cleft and surrounding extracellular space [280, 281]. Recent evidence from Drosophila further suggests that glial lipid metabolism can modulate circadian behavior through glutamate handling. Loss of ceramide phosphoethanolamine or sphingomyelin in astrocyte‐like glia disrupts circadian rhythmicity and shortens lifespan, at least partly through EAAT1‐mediated excitotoxicity, whereas maintenance of ceramide phosphoethanolamine/sphingomyelin improves circadian rhythms and significantly prolongs lifespan [282]. These findings expand the concept of astrocyte metabolic reprogramming from energy and redox metabolism to lipid‐dependent circadian‐metabolic coupling, suggesting that time‐dependent regulation of astrocytic glutamate transport and lipid homeostasis may influence neuroinflammatory vulnerability and disease progression.

Emerging research also highlights the significant roles of metabolic pathways like NAD+ metabolism and sphingosine‐1‐phosphate signaling in regulating astrocyte pro‐inflammatory gene expression and neurotoxicity [283, 284, 285, 286]. Therefore, metabolic reprogramming is not a passive consequence of reactivity but an active core element driving its phenotypic and functional heterogeneity. Targeting specific metabolic checkpoints‐such as restoring lactate metabolism, enhancing glutamate uptake capacity, or correcting mitochondrial function‐holds promise for reversing astrocytes from a pathogenic metabolic mode to a neuroprotection‐supportive mode by reshaping their metabolic state, offering promising therapeutic strategies for treating neuroinflammatory diseases.

## 6 Therapeutic Strategies Targeting Astrocytes and Future Directions

### 6.1 Current Therapeutic Strategies and Challenges

Current therapeutic strategies targeting astrocytes have achieved progress across multiple fronts, primarily focusing on metabolic regulation, intervention in intercellular interactions, targeting key receptors and transporters, and modulation of the gut‐brain axis. Metabolically, inhibitors of glucosylceramide synthase like Miglustat effectively suppress the pathogenic immunometabolic activity of astrocytes by blocking the cPLA2‐MAVS signaling pathway and have shown therapeutic potential in experimental autoimmune encephalomyelitis models [53, 287]. Meanwhile, the mitochondria‐targeted antioxidant XJB‐5‐131 highlights the neuroprotective value of metabolic intervention by mitigating oxidative stress‐mediated neuronal damage [138, 288]. Regarding intercellular interactions, inhibition of axon guidance molecules, such as the EPHB3‐ephrinB3 and plexinB1/2‐semaphorin4D pathways, has been shown to alleviate pathogenic astrocyte‐microglia crosstalk, providing potential intervention targets for diseases such as MS [72, 139, 176, 289]. Furthermore, the astrocyte‐expressed glutamate transporter EAAT2/GLT‐1 is an important therapeutic target; ceftriaxone can increase GLT‐1/EAAT2 expression and has shown neuroprotective effects in preclinical models of ALS and AD [290, 291]. Sphingosine‐1‐phosphate receptor modulation also has translational relevance: fingolimod suppressed pathogenic astrocyte activation in a chronic‐progressive EAE model, whereas siponimod reduced disability progression in a phase 3 SPMS trial [292, 293].

However, existing strategies still face numerous challenges. Efficient penetration of the BBB remains a major bottleneck for drug delivery. Although novel carrier systems like nanoparticles and dendritic polymers have shown astrocyte‐specific targeting potential in preclinical studies, in vivo stability, biodistribution, and long‐term safety require further validation [294, 295, 296]. Concurrently, the high heterogeneity of astrocytes and their phenotypic plasticity across different disease stages (e.g., the dynamic spectrum of reactive astrocyte states) often render single‐target interventions of limited efficacy, potentially even causing adverse effects by disrupting physiological functions. Moreover, several failures in clinical trials (e.g., Laquinimod for HD and anti‐SEMA4D antibody therapies, which showed effects on some secondary endpoints but failed to meet primary endpoints) [297, 298, 299, 300, 301] serve as warnings that the complexity of disease mechanisms, patient heterogeneity, and the critical timing of intervention remain key challenges for clinical translation.

### 6.2 Emerging Therapeutic Directions

With advancements in molecular biology and biotechnology, a range of emerging therapeutic directions is injecting new momentum into astrocyte‐targeted interventions. The fields of gene therapy and cellular reprogramming are particularly promising: The CRISPR/Cas9 system enables efficient editing of specific genes (e.g., STAT3, APOE4, TREK‐1) in astrocytes, providing precise means to correct pathogenic signaling pathways, enhance Aβ clearance capacity, and restore ionic homeostasis [9, 73, 78, 302, 303]. Direct transdifferentiation of astrocytes into functional neurons (via targeting PTBP1 or overexpressing neurogenic factors like NEUROD1) has successfully reversed motor deficits in experimental models of PD and HD, demonstrating immense potential for regenerative medicine, despite ongoing debates regarding cell‐of‐origin and conversion efficiency [304, 305, 306, 307].

Specific modulation of detrimental reactive astrocyte programs represents another therapeutic frontier. Preclinical strategies including HDAC inhibition with LASSBio‐1911 [308], modulation of astrocytic GPCR signaling [309, 310, 311], and mTOR inhibition [312, 313] can alter astrocyte reactivity and inflammatory output in experimental models. NLRP3‐related neuroinflammatory pathways are also being investigated preclinically as potential targets for limiting injury‐associated astrocyte activation [314]. Glucagon‐like peptide‐1 receptor agonists have additionally been explored for neuroprotective and anti‐inflammatory effects. NLY01 reached a completed phase 2 trial in early untreated PD; however, it did not improve the primary motor or non‐motor outcomes compared with placebo [315].

Engineered probiotics and gut‐brain axis modulation represent a novel therapeutic paradigm [316, 317]: Probiotic strains designed via synthetic biology can be directed to produce neuroactive metabolites (e.g., tryptophan‐derived AHR agonists) [191] or modulate the functional state of peripheral immune cells (e.g., natural killer cells) [73], thereby remotely regulating astrocyte inflammatory activity and the central immune environment, opening non‐invasive avenues for systemically intervening in neuroinflammation.

The potential of these emerging therapeutic directions (gene therapy, A1 phenotype regulation, probiotics, etc.) has been initially verified in numerous preclinical studies and some early clinical trials, as summarized in Table 2.

**TABLE 2:** Representative preclinical animal experiments and clinical trials targeting astrocytes.

Target Pathway	Drug/Method	Type	Disease	Findings/Outcomes	Reference
cPLA2‐MAVS Axis	Miglustat	Preclinical Animal Experiment	EAE	Reduce neuroinflammation and demyelination	[53, 287]
S1P Receptor	Fingolimod, Siponimod	Preclinical mechanistic study; Phase 3 clinical trial	Chronic‐progressive EAE; SPMS	Fingolimod suppressed pathogenic astrocyte activation in chronic‐progressive EAE; siponimod reduced the risk of disability progression in SPMS	[292, 293]
EAAT2 (GLT‐1)	Ceftriaxone	Preclinical Animal Experiment	AD, ALS	Upregulates GLT‐1/EAAT2; delays disease‐related functional decline in ALS models and improves pathology and cognition in AD models	[290, 291]
JAK/STAT3 Pathway	Multiple Inhibitors	Preclinical Animal Experiment	AD	Reduce Aβ plaque burden and improve cognition	[84]
MAFG/MAT2α	Conditional Knockout of MAFG/MAT2α	Preclinical Animal Experiment	EAE	Significantly reduce disease severity	[20, 255]
SEMA4D	Pepinemab (VX15/2503)	Clinical Trial (Did Not Meet Primary Endpoint)	HD	Show partial improvement in some cognitive endpoints but did not meet primary endpoint	[298]
NLRP3 Inflammasome	Astrocyte‐specific NLRP3 knockout	Preclinical Animal Experiment	Mild TBI	Reduce neuroinflammation and depression‐like behaviors	[314]
GLP‐1 Receptor	NLY01	Phase 2 clinical trial (completed)	PD	Completed phase 2 clinical trial; NLY01 did not significantly improve the primary motor or non‐motor outcomes compared with placebo	[315]

### 6.3 Future Research Directions and Technological Advances

Looking ahead, astrocyte‐directed research should prioritize functionally validated, disease‐, region‐, and stage‐specific states rather than marker‐defined categories alone. Integrating single‐cell and single‐nucleus transcriptomics with spatial transcriptomics, epigenomics, proteomics, and functional perturbation can refine astrocyte state maps and identify more selective therapeutic targets [318, 319, 320, 321]. These state maps may support the rational development of astrocyte‐directed delivery systems and molecular interventions; however, cell‐type specificity, blood–brain barrier penetration, biodistribution, and long‐term safety remain major translational constraints.

Therapeutic design should also account for disease stage and the context‐dependent protective and harmful functions of reactive astrocytes [322, 323, 324]. Rather than prescribing a universal “A1‐to‐A2” switch, future studies should test stage‐adaptive modulation of defined inflammatory, metabolic, and epigenetic programs. Clinical translation will require validated biomarkers and human‐relevant models. Current neuroimaging and fluid biomarkers can report neuroinflammatory or disease activity but are not sufficiently astrocyte‐specific; in particular, TSPO PET reflects signals from multiple cell types and should not be interpreted as a specific real‐time measure of astrocyte activity. Human iPSC‐derived astrocytes and related multicellular models provide complementary platforms for mechanistic testing and individualized drug screening [325, 326, 327]. Gene editing, nanodelivery, and cellular reprogramming remain promising but require rigorous assessment of target specificity, durability, off‐target effects, biodistribution, and long‐term safety.

## 7 Conclusion and Perspectives

This review highlights the central role of astrocytes in neuroinflammatory responses across neurological disorders. Astrocytes are no longer regarded merely as passive support cells, but as active regulators of the CNS immune network. Under pathological conditions, they undergo dynamic and heterogeneous reactive remodeling that cannot be fully explained by the traditional A1/A2 dichotomy. Accumulating single‐cell, spatial transcriptomic, and functional studies indicate that astrocyte reactivity represents a heterogeneous and dynamic continuum shaped by disease type, brain region, pathological stage, and intercellular crosstalk [318, 319, 320]. In AD, PD, ALS, HD, CNS injury, and CNS infection, astrocytes influence disease progression through inflammatory mediator release, metabolic reprogramming, disruption of ionic and neurotransmitter homeostasis, and extensive communication with microglia, neurons, oligodendrocytes, endothelial cells, and peripheral immune cells [24, 321, 328].

Despite these advances, several key questions remain unresolved. First, it is still unclear how disease‐relevant astrocyte states should be defined. Marker‐based classification alone is insufficient, as astrocyte phenotypes are shaped by disease type, anatomical region, pathological stage, and cellular crosstalk [9, 322, 329]. Future studies should integrate single‐cell transcriptomics, spatial omics, epigenomics, proteomics, metabolomics, and functional perturbation approach to establish disease‐, region‐, and stage‐specific astrocyte state maps. Second, the dual role of astrocytes creates a major therapeutic challenge. Astrocytes can amplify neuroinflammation through cytokine release, complement activation, excitotoxicity, oxidative stress, and immune cell recruitment, but they may also preserve blood‐brain barrier integrity, support neuronal metabolism, restrict tissue damage, and promote repair [28, 323, 324]. Therefore, future therapeutic strategies should move beyond broad suppression of astrogliosis and instead selectively target detrimental astrocyte programs while preserving protective functions.

Furthermore, clinical translation requires more precise models, biomarkers, and delivery systems. Human induced pluripotent stem cell‐derived astrocytes, brain organoids, organ‐on‐chip platforms, and spatially resolved co‐culture systems may better model human astrocyte interactions. Imaging signals, cerebrospinal fluid or blood‐based inflammatory markers, and astrocyte‐derived extracellular vesicles may support patient stratification and treatment monitoring [325, 326, 327]. Meanwhile, nanocarriers, viral vectors, antibody–drug conjugates, and gene‐editing tools require further validation for specificity, blood–brain barrier penetration, biodistribution, and long‐term safety. Importantly, astrocyte‐targeted interventions should also consider disease stage. Astrocyte programs that are harmful during acute inflammation may differ from those required for repair during subacute or chronic phases [28, 66]. Thus, stage‐adaptive strategies may be more effective than uniform intervention approaches.

Overall, astrocytes should be viewed not merely as downstream responders to CNS pathology, but as dynamic regulators of neuroimmune homeostasis, disease progression, and tissue repair [24, 330]. Future research should focus on defining functionally validated astrocyte states, clarifying causal interactions within the CNS immune network, and developing biomarker‐guided, stage‐specific therapeutic strategies. Such efforts may provide new opportunities for disease modification in neuroinflammatory and neurodegenerative disorders.

## Author Contributions

**Shijun Peng**: conceptualization, methodology, writing – original draft, visualization. **Lepeng Wang**: investigation, methodology, writing – review & editing. **Jia Ouyang**: supervision, funding acquisition, writing – review & editing. **Ruen Liu**: administration, funding acquisition, writing – review & editing. All authors have read and approved the final manuscript.

## Funding

This review was supported by the funding from the National Key Research and Development Program of China (grant no.: 2019YFE0117100), the National Natural Science Foundation of China (grant nos.: 82104603 and 82074174). This work was also supported by the Beijing Natural Science Foundation(grant no.: 7262216).

## Ethics Statement

The authors have nothing to report.

## Conflicts of Interest

The authors declare no conflicts of interest.

## Data Availability

The authors have nothing to report.
